# Visualising the voices of nursing: a co-designed video capturing the lived experiences of nurses in Northern Ireland during the COVID-19 pandemic

**DOI:** 10.1186/s12912-025-02881-9

**Published:** 2025-03-03

**Authors:** Carolyn Blair, Dame Anne Marie Rafferty, Paul Murphy, Michael Brown, Karen Bowes, Ruth Thompson, Joanne Reid

**Affiliations:** 1 School of Nursing and Midwifery, Queens University Belfast, Belfast, UK; 2 Florence Nightingale Faculty of Nursing, Midwifery & Palliative Care, Kings College London, London, UK; 3 School of Arts, English, and Languages, Queens University Belfast, Belfast, UK; 4 Royal College of Nursing, Belfast, UK

**Keywords:** Nurses, COVID-19, Verbatim, Storytelling, Lived experience, Monologues, Video, Systematic text condensation

## Abstract

**Background:**

Nurses were at the forefront of managing the COVID-19 pandemic. In response, the Royal College of Nursing in Northern Ireland commissioned a longitudinal qualitative survey using the Cognitive Edge SenseMaker^®^ tool to capture nurses’ experiences of delivering care from April 2020 to March 2021.

**Aim:**

To explore the effect of a co-designed video based on the findings of SenseMaker^®^, of the lived experience of nurses in Northern Ireland during the 2020/2021 global pandemic.

**Method:**

Quotes were selected from the SenseMaker^®^ report of nurses’ (*n* = 676) which conveyed the experiences of nurses during COVID-19. Three co-design workshops were conducted. The first covering the plan for extraction of data from the SenseMaker^®^ report, the second content development and script writing and the third covering feedback and revisions. The video was filmed and edited in the Drama Studies, School of Arts, English and Languages, Queen’s University Belfast. The live launch event took place in the Royal College of Nursing conference venue on 8th February 2024. Data to gauge the effect of the video were gathered via audience participation, MS Teams Version 1.7. chat participation, Mentimeter poll and MS forms survey. The link for the video and survey was accessible via the School of Nursing and Midwifery, Queens University Belfast website after the launch event. The findings were analysed using systematic text condensation using NVivo version 1.6. The study was approved by the Faculty of Medicine, Health and Life Sciences research committee at Queen’s University Belfast following peer review (REC Reference: MHLS 23_100).

**Results:**

Twenty-eight participants completed the survey; 30 participants attended the live event. Overall, 93% (*n* = 26) of participants confirmed that the video-based monologues effectively conveyed the emotional perspective and lived experiences of nurses during the COVID-19 pandemic. Four themes emerged - personal reflection and emotional effect, connection and solidarity amidst disappointment, moral injury and resentment, lessons learned and the need for change.

**Conclusion:**

Wider dissemination of the co-created video-based narratives, with focus on advocacy to policy makers, is needed to prioritise the emotional well-being of nurses and other professionals. There is potential in using video-based monologies to facilitate positive change and better support for professionals, including nursing students in future crises. Further research is needed to assess the broader effect of such healthcare-related research methodologies.

**Supplementary Information:**

The online version contains supplementary material available at 10.1186/s12912-025-02881-9.

## Background

On 30th January 2020, the World Health Organisation (WHO) declared that the coronavirus disease 2019 (COVID-19) outbreak was an international public health emergency [1]. In 2020 and 2021 it is estimated that there were 14.83 million excess deaths globally [2]. Nurses were at the global forefront of providing care and support throughout the pandemic, all of whom faced the occupational risk of becoming infected with COVID-19, and at worst, death [3–7]. During the pandemic, nurses were working under immense physical and psychological pressure making critical decisions while treating and caring for an exponentially growing number of acutely ill patients [7].

Multiple studies have sought to record the psychological effects of the pandemic on the nursing and midwifery workforce, for example a longitudinal UK based survey gathered valuable quantitative data evidencing the adverse psychological effects of the work environment [8]. In the impact of COVID-19 on nurses (ICON) survey, which included a large sample of nurses and midwives (*n* = 3299) at an early stage of the pandemic in April 2020, findings revealed that a failure to meet nurses needs to be safe at work significantly impacted on morale [9]. In a further qualitative study authors conducted interviews with 50 nurses, midwives and students working in a range of settings during the COVID-19 pandemic which provided qualitative data on the effects of the COVID-19 pandemic on nurses’ psychological wellbeing [10]. Findings reported the experiences of the traumatic and distressing working practices nurses encountered during COVID-19 and highlighted the organisational issues that significantly affected their psychological wellbeing. Maben et al.’s [11] realist review provided a strong rationale for raising awareness and improving the systemic working conditions to improve psychological well-being for nurses and midwives. This review recommends that interventions should be co-designed with front-line staff and staff experts by experience, and tailored where possible to local, organisational and workforce needs [11]. Like other reviews based on experiences of COVID-19 for nurses, the data in most of the articles included in this review was collected between January and May 2020 [12] which does give a window into early experiences of COVID-19, however, does not give a longitudinal perspective over the duration of the pandemic.

The SenseMaker^®^ report was commissioned by the Royal College of Nursing (RCN) Northern Ireland (NI), the final report of which is within the public domain [13]. This survey is unique in that it longitudinally gathered qualitative data (03/2020–03/2021) from a large anonymous sample of nurses (*n* = 676). To do this it used the Cognitive Edge SenseMaker^®^ tool, which is a recognised instrument used to capture and make sense of people’s attitudes, perceptions and experiences [14]. This enabled the collection and analysis of qualitative narratives, allowing nurses to share stories of their recent experiences [13],. Data were collected through a dedicated project site accessible via web or phone browsers, with the URL distributed directly by RCN to nurses across NI [13]. The SenseMaker^®^ report provides deep insights into the complex issues faced by nurses, contextualised through their self-interpreted stories during the pandemic [13].

Using videos to showcase verbatim narratives is known to be a powerful medium to share research findings with a wide international audience [15]. Communication research suggests that people integrate both factual and narrative information when making decisions as narrative information affects choices directly through emotional engagement and indirectly through cognitive processing [16, 17]. These insights highlight the opportunity afforded by the findings of the publicly available SenseMaker^®^ report, to reproduce these compelling verbatim narratives through the medium of storytelling via video-monologues [15, 18].

The verbatim stories presented in the SenseMaker^®^ report bring to light the challenges faced by nurses, the emotional and physical toll at all stages of the pandemic [13]. However, despite the significant stressors which impacted heavily on the mental health and wellbeing of nurses, as illustrated through the narratives in the SenseMaker^®^ report [13] and evidence from other research [3–12], their experiences remain under-recognised. Consequently, there is an opportunity and need to find accessible and engaging ways to raise awareness and educate nurses, the wider public, health services and policy makers of the consequences of the lived experiences of nurses.

## Methods

### Aim and objectives

#### Study aim

To explore the effect of a co-designed video based on the findings of SenseMaker^®^: the lived experience of nursing in Northern Ireland during a pandemic 2020/2021.

#### Objectives

- To co-design a video monologue based on the SenseMaker^®^ report.
- To explore the perceived congruence of the content from the perspective of HCPs and the public.
- To assess whether video-based monologues are a useful way of disseminating qualitative healthcare related research.

### Methods

**Fig. 1:**
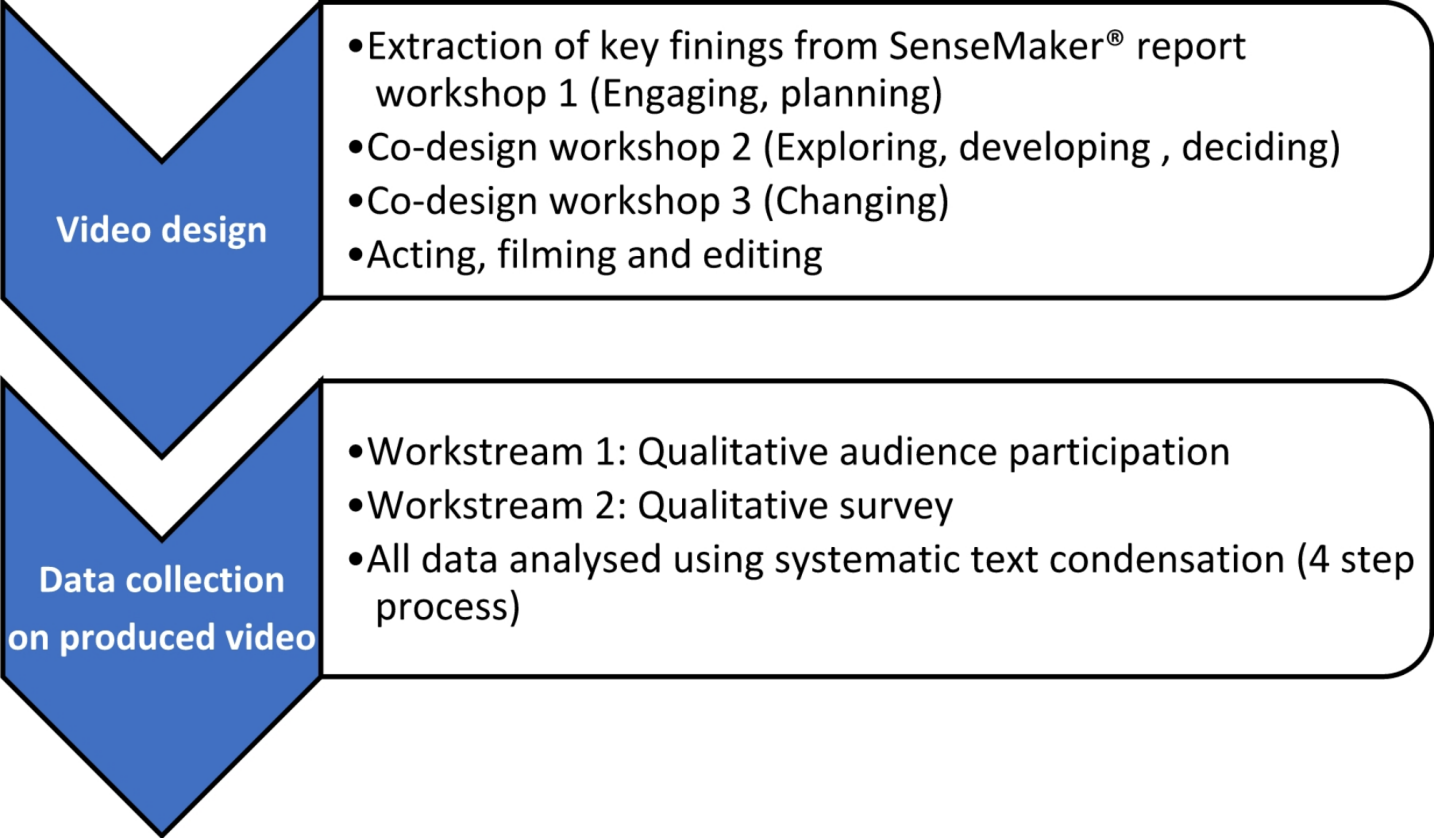
Overview of methodology

The methods section presented below has two parts, see Fig. 1:

1. Co-design of the video.
2. Data collection on produced video.

#### Methodology for the co-design of the video

The appropriateness of using co-design in the development of such a video is highlighted within the literature [19]. Developing the video aligned with Boyd’s [20] six steps of co-design, including Engaging, Planning (workshop 1), Exploring, Developing, Deciding (workshop 2), and Changing (workshop 3). The script design was an iterative development process and nurses were involved at every stage of this process.

##### Engaging, planning (workshop 1) - *Extraction of key findings from the SenseMaker*^®^ [13] report

A group of six members were purposively selected by JR on the basis of their experiential and disciplinary diversity in nursing, and formed the co-design team. The co-design process involved five registered nurses (JR, MB, AMR, KB, ZO), and one student nurse (TM). An expert in dramaturgy (PM) and healthcare researcher (CB) facilitated the group but were also actively involved in the co-design process. The purpose of the co-design team was to enable a variety of relevant experiences and preferences to be integrated into the co-design of the video. Post invitation to the group an engagement and planning workshop (online– 1 h) was held with the six members of the co-design team and two facilitators to decide how the data would be extracted from the publicly available SenseMaker^®^ [13] report for the video-based monologues to most accurately illustrate the key findings. The team agreed that PM, an academic who had significant experience in dramaturgy (defined as the theory and practice of dramatic composition) and arts-based healthcare projects, would be best placed to extract a preliminary list of quotations from the SenseMaker^®^ report. The team agreed that the following four steps were essential to ensure accuracy in the initial extraction of quotations:

1. Rigorous review of the SenseMaker^®^ report to identify central themes and significant insights from the sections ‘Emotional overview of the year’ and ‘Key themes’. This process aimed to highlight key challenges, coping strategies, and emotional effects identified in the report, to inform and support the nursing community.
2. Quotation selection: Selecting quotations that vividly illustrated these themes, focusing on the emotional and experiential aspects of the nurses’ stories.
3. Thematic categorisation: Categorising quotations into four thematic areas: professional challenges, coping strategies, and emotional effects to ensure comprehensive coverage of the report’s findings.
4. Second checking: To ensure that the selected quotations accurately reflected the overall findings of the report, the co-design team nominated two participants (KB, AMR) who had in-depth knowledge of the SenseMaker^®^ report to assess whether the selected quotes were an accurate reflection of the report.

##### Exploring, developing, deciding (workshop 2) - content development and script writing

In this workshop all members of the co-design team contributed to content development and script writing (in-person– 2 h). The selected quotations from the SenseMaker^®^ report were presented to the co-design team, each team member had a copy of the selected list and read a quotation After each theme, the quotations were discussed and quotations which were deemed irrelevant or repetitive removed consistently ensuring the content represented nurses’ experiences accurately as portrayed within the SenseMaker^®^ report. A script was developed and quotations put in an order which was deemed to tell the story of nurses experiences through the pandemic with intensive input from the co-design team.

##### Changing (workshop 3)– feedback and revisions

This workshop was conducted online (1 h), the draft script was sent to team members prior to the meeting. In this workshop the quotations were read in the proposed order that they would appear in the video, this was reviewed with the co-design team and necessary revisions made based on feedback.

##### Acting, filming and editing

The untrained actors involved were the five registered nurses (JR, MB, AMR, KB, ZO) and one student nurse (TM). Performances by untrained actors were intentional to emphasise the universal relatability and human aspect of the narratives. The video monologues were filmed and edited in the School of Drama, QUB and performed by attendees involved in the co-design workshop, all of whom were registered nurses. Editing of the video continued with the full involvement of the co-design team.

#### Methodology for data collection

##### Study design

The study used qualitative methods comprising two workstreams (WS):

WS1: Qualitative audience participation responses using an initial mentimeter question prompt (https://www.mentimeter.com a free-to-use interactive presentation platform for hosting Live Q&A sessions and polling). The mentimeter prompt was researcher devised based on encouraging participants to convey their initial thoughts about the video and promote interactivity in the audience discussion. Further questions were guided by audience participation via live responses at the event and MS Teams (Version 1.7) chat function.

WS2: Qualitative survey explored the video in greater depth. The survey gathered data on broader experiences and issues relating to the experience of nurses through the COVID-19 pandemic providing insight into these wider experiences and concerns.

##### Sample and recruitment

Participants were recruited using non-probability convenience sampling. The RCN NI sent an invitation email and participant information sheet to all members which informed potential participants of the process regarding consent (see further details in Ethical considerations and informed consent). Through this email, participants wishing to attend the live (Workstream 1) or online (Workstream 2) launch event were invited to register by sending an email back to the corporate events team at RCN NI. To ensure that members of the public were also aware of the launch event and study, this was promoted by collaborators in Queen’s University Belfast (QUB) and RCN NI using social media platforms such as X, Instagram, and Facebook. If potential attendees saw the flyer via social media, wished to attend, and contacted the corporate events team at RCN, the corporate events team sent the information to ensure potential participants were fully aware of the research study and the process regarding consent. The inclusion criteria were intentionally broad to ensure inclusivity of all adults over the age of 18 who had an interest in the experience of nurses through the COVID-19 pandemic. Please see details in Table 1.

**Table 1:** Inclusion and exclusion criteria

Inclusion criteria	Exclusion criteria
Adult (aged > 18 years)	< 18 years
Has an interest in the experience of nurses through the COVID-19 pandemic	No interest in the experience of nurses through the COVID-19 pandemic
Able to read, use email, a computer and/or phone	Not able to use a video conferencing tool*
Able to use a video conferencing tool*	Unable to read, use email, a computer and/or phone

##### Data collection

###### Live launch event (Workstream 1)

The live event took place in the Royal College of Nursing RCN NI conference venue on 8th February 2024. Audience participation was prompted using an initial mentimeter question prompt which was also accessible to those who joined online and was used to ease the participants into the audience discussion. The audience discussion was directed by the responses to the mentimeter question and audience participation. Flexibility was an essential element of this qualitative design, allowing the audience participation techniques to be adapted to the individual participant who wished to share their thoughts. The facilitator of this process (MB) was an experienced qualitative researcher from the research team.

###### Survey (Workstream 2)

The qualitative survey explored the effect of the video in greater depth and was co-created by the research team (Please see supplementary document 1). The survey gathered data on broader experiences and issues relating to the experience of nurses through the COVID-19 pandemic, providing insight into these wider concerns. The qualitative survey collected demographic information (for example, age, nationality, healthcare discipline). It also contained topic questions relating to the study, concluding with closing questions providing participants with the opportunity to share anything else, i.e., raise any issues or concerns [21]. Questions were based on the experience, feelings, and knowledge of the participants as recommended by Patton et al. [22]. For example, “Has your understanding.?“, “In your opinion.?“, “What do you think.?” Probe questions were used throughout the survey as appropriate, e.g., “please explain.” This was considered good practice by Morse and Field [23] to avoid the researcher losing vital information. The link for the video and survey was accessible via the School of Nursing and Midwifery, QUB website after the launch event. All those attending the live launch event were all invited to complete the survey.

##### Ethical considerations and informed consent

The methodology for this study and all supporting documents were approved by the Faculty of Medicine, Health and Life Sciences (MHLS) research committee at QUB following peer review (REC Reference: MHLS 23_100). Fundamental principles of good practice including informed consent, voluntary participation, confidentiality and data protection procedures were applied as a minimum standard in the study [24]. Informed consent to participate was obtained from all participants. Participation in this research project was voluntary. Attendance lists and email addresses were stored with the RCN NI corporate events team, which was not accessible to the QUB research team. Considering that identifiable information (i.e. email addresses or names) would not be collected, participants were informed through the Participant Information Sheet that by agreeing to attend the event (either in person or online) this signified consent for the research team to collect and use the information they provided for the research project. All participants attending the event were sent a Participant Information Sheet by the RCN NI corporate events team, via email, prior to the event. They were also informed that they were not obligated to participate in the audience discussion vocally if attending the event or via the live chat on MS Teams (Version 1.7). Additionally, all participants including those who did not attend the event were informed again via the survey that no identifiable data would be collected and if they submitted the survey, this would signify consent to participate in the research study. The data was stored on encrypted computers and was only accessible by named researchers in the study, in line with General Data Protection Regulations [25]. The research team were also cognisant of the fact that viewing a video of nurses’ experiences during COVID-19 had the potential to induce or exacerbate emotional distress, therefore a distress protocol was adhered to [26].

##### Data analysis

This qualitative methods study used two methods of data collection, workstream 1 (W1) and workstream 2 (W2) to enable a deeper understanding of the broader themes emerging from this work [27]. Intra-paradigmatic mixing is deemed to be an effective method to address complex research problems. This was particularly relevant in this research to ensure that all voices are heard via their preferred platform. A comprehensive cross thematic analysis was undertaken from the qualitative data from the completed surveys, the transcript of the live audience participation, the transcript of the chat function from MS Teams (Version 1.7) and the response to the mentimeter prompt (W1 and W2). These responses were coded using systematic text condensation (STC) [28]. STC is a descriptive and explorative method for thematic cross-case analysis of different types of qualitative data such as audience participation and analysis of written text [28]. This method was therefore deemed appropriate for the analysis of the data obtained from W1 and W2. STC involved four steps which we used in terms of data analysis (1) total impression -chaos to themes; (2) identifying and sorting meaning units–from themes to codes; (3) condensation from code to meaning and (4) synthesising - from condensation to descriptions and concepts [28]. The approach enabled cross case synthesis to develop expressive category statements elucidated from the analysis of the participants responses [28]. NVivo version 1.6 [29] was used to assist the management and sorting of data of W1 and W2 which was analysed using the four steps from STC [28]. All data excerpts were read and coded by two members of the research team (JR and CB) prior to following the four steps. Study findings were reported in line with Standards for Reporting Qualitative Research [30].

##### Rigour

As detailed within this methodology section, the data collection and analysis were undertaken through transparent methodological processes to optimise rigour within this study. Triangulating selected sections from the SenseMaker^®^ report with: nurses within our research team who worked as front line staff during the COVID-19 pandemic (MB, KB, RT); and with co-design workshop participants who had worked as either student nurses or registered nurses during this time, ensured an accurate reflection of both data from the SenseMaker^®^ report and the lived experience of nurses during the pandemic, which were used within the co-designed video. As detailed in the data analysis section, two researchers (JR and CB) independently analysed the qualitative data to ensure it was accurately represented within the analysis presented. The use of triangulation, meaning confirmation and independent analysis to draw valid conclusion from the data substantiates the credibility of this research [31]. Additionally, transferability is augmented through the details of sampling, recruitment and timeframe of data collection presented within the methodology section( [32].

## Results

### Demographic profile of participants

Thirty participants attended the live event *n* = 13 in person, *n* = 17 online. A total of *n* = 28 participants completed the survey, *n* = 22 participants who attended the live event completed the survey an additional *n* = 6 who could not attend watched the video and completed the survey at their convenience. Their information is presented in Table 2 below.

**Table 2:** Demographic information of participants who completed the survey

Demographic	
**Gender**	*N* = 24 (86%) Female
	*N* = 4 (14%) Male
**Age**	*N* = 1 (4%) 18–30 years
	*N* = 3 (11%) 31–43 years
	*N* = 15 (54%) 44–56 years
	*N* = 9(32%) 57 + years
**Country of origin**	*N* = 6(21%) United Kingdom
	*N* = 1(4%) Ghana
	*N* = 8 (29%) did not disclose county of origin
**Occupation and workplace**	*N* = 20 (72%) registered nurses *Workplace* *N* = 7 (25%) higher education institute *N* = 4 (15%) community *N* = 3 (11%) hospital setting *N* = 1 (4%) self-employed *N* = 1 (4%) care home *N* = 1 (4%) hospice *N* = 1 (4%) palliative care charity *N* = 1 (4%) retired *N* = 1 (4%) RCN
	Other occupations workplace *N* = 3 (11%) higher education institute in healthcare research *N* = 3 (11%) participants did not respond to this question *N* = 1 (4%) stated that they were a member of the public and had an interest in this area *N* = 1 (7%) in a care home.

### Themes from systematic text condensation

Using the qualitative data from the completed surveys, the transcript of the live audience participation, the transcript of the chat function from MS Teams (Version 1.7) and the response to the mentimeter prompt (W1 and W2), four themes which illustrated the effect of the video-based monologues were identified - personal reflection and emotional effect, connection and solidarity amidst disappointment, moral injury and resentment and lessons learned and the need for change.

#### Personal reflection and emotional effect

The video conveyed the stress, emotional toll, and challenges faced by nurses during the pandemic, bringing their experiences to life and highlighting the emotional effect on their values, beliefs, and personal lives. The emotional effect of the pandemic on participants was clear through the mentimeter response, the survey and the audience discussion.

*I could feel the hurt*,* the sadness*,* the loneliness*,* the fear and uncertainty and I related to it (Survey participant 21)*.

Participants indicated that there were immersed in a state of reflection following watching the video. The mention of losses, heartbreak, and deaths emphasises the profound toll and lasting impression that COVID-19 had on both patients and healthcare workers, which was re-experienced through watching the video.

*A reminder of what we had come through [as nurses]*,* the losses the heartbreak and the deaths. Something I will never forget. (Survey participant 16)*

The video also prompted a wider recognition from those who were not frontline workers during the pandemic, as participants reflected on the newfound understanding and appreciation for the role of healthcare workers, particularly in times of crisis.

*The narratives of the various nursing staff were extremely moving and incredibly real. I’m not a nurse*,* but this really brought the issues that nurses were facing to life for me. (Survey participant 14)*

The findings illustrate that the video was a catalyst for participants to recognise the sacrifice and selflessness demonstrated by nurse and other health professionals as a depth of dedication and commitment to their profession and the people they served during the pandemic.

*It really made me understand the importance of health workers*,* how helpful they were*,* even at the detriment of their own life. (Survey participant 23)*

Participants indicated the importance of remembering and acknowledging the challenges, experiences, and sacrifices made during the COVID-19 pandemic, suggesting that the video serves as a medium through which these memories are preserved and honoured. The video triggered recollections of past experiences, likely related to the challenges and hardships endured during the pandemic. Participants emphasised the importance of remembering and acknowledging the difficulties faced during that time and a need to ensure that these experiences are not overlooked or erased from memory.

Brought back memories and ensured what was endured is not forgotten. (Survey participant 11)

Overall, the power of the video was emphasised in evoking memories and reinforcing the significance of preserving collective memory. The video was noted to serve as a time capsule containing memories, experiences, and events from that period underscoring the importance of remembering and acknowledging the events and challenges faced during this time.

Capsule in time to depict and not forget this period in time (Survey participant 26)

Overall, participants expressed their recognition of the video’s role in preserving and commemorating a very significant historical period.

#### Connection and solidarity amidst disappointment

Watching the video fostered a sense of connection and solidarity among nursing colleagues, and served as a reminder that they were not alone in their experiences and encouraged support for one another. The mention of shared values and beliefs among nurses suggested a sense of connection and solidarity within the nursing community which helped them to survive the emotional effect of working through COVID-19. The hardships endured were not only acknowledged but also deeply felt by participants, as the stories depicted resonated with their own and their colleagues’ experiences during the pandemic.

*Watching the video made me recall some of the events and personal stories that happened to me and my colleagues during Covid 19. Some of the points highlighted by the individuals in the video highlighted that many nurses have similar values and beliefs*,* and the stories drive home some of the hardships nurses endured during Covid. (Survey participant 2)*

Following watching the video, many participants with a nursing background suggested there was a sense of cohesion and solidarity amongst their colleagues despite the intensity of what they were facing. The video served as a reminder of the value of teamwork working through the multifaceted challenges posed by the pandemic. However, for many there was also an underlying sense of resentment which was linked to the perceived expectations and under appreciation from those who were not frontline workers.

*There were more extremes… in that patients were dying and there was very little the doctors or nurses could do for the patients. There was on the other hand a great sense of teamwork. Outside of work people were being isolated and getting paid to stay at home and stay safe*,* get fit and paint their houses while nurses were expected to go into work and put their lives at risk each day. (Survey participant 2)*

After watching the video, for several participants, there was a realisation that circumstances were worse in some areas, they signalled shock and disappointment at what they perceived as a failure of leadership. Feelings of frustration and disillusionment with the management’s response to the crisis was clear, illustrating the video’s power in uncovering stories which participants were previously unaware of.

*I feel teams were drawn together and did their best*,* but I hadn’t realised how bad it was in some places The apparent lack of support from managers was shocking. (Survey participant 19)*

Having watched the video, participants emphasised the importance of validation and recognition of nurses’ emotions within the context of the nursing profession during the pandemic. Participants expressed how the video underscored the fundamental need for their feelings to be acknowledged and affirmed by their peers, social support and affirmation was essential in coping with the emotional challenges they faced.

*The [video highlighted the] need to have my feelings validated by my colleagues. (Survey participant 16)*

For the majority, the video underlined the need to prioritise the emotional well-being of nurses and other health professionals with a sense of advocacy and empowerment, suggesting a commitment to ensuring that nurses’ voices and emotions are heard and respected. Participants felt the video helped to validate what many personally experienced during the pandemic, particularly considering perceived past neglect or oversight.

*[most important benefit of watching the video was…] Validation of nurses’ feelings. We were overlooked then we can’t be overlooked now (Survey participant 18)*.

#### Moral injury and resentment

Following watching the video, participants reflected on how the treatment of nurses post-COVID has inflicted more damage on their morale and sense of value than the pandemic itself. This illustrates the power of the video-based monologues in helping nurses and other professionals to share their personal experience in reflection of the content of the video. Furthermore, there was a sense of moral indignation that despite their dedication and sacrifices during the crisis, the video prompted several participants with a nursing background to share that they felt undervalued, unappreciated and resentful in the aftermath.

*I think how nurses were treated after COVID has done us more damage than COVID did to our profession in terms of morale and in terms of feeling valued and in terms of wanting to keep giving. (Audience Participant 1)*

After the video some participants reflected on the challenges of reintegrating into society and readjusting to normalcy after the pandemic. The comparison to soldiers returning from war, emphasised nurses’ feelings of emotional loneliness in their interactions with those who did not understand what it was like as a nurse or health professional during the pandemic. This was compounded by the sense of their sacrifices being forgotten.

*The post-Covid experience is reminding me of war veterans*,* in particular when they came back from World War I*,* from the trenches*,* from a place that nobody knew what it was like… they came back and they had to sort of re-enter City Street and very quickly… they’re very soon forgotten and told to sort of like*,* oh*,* just get on with it. (Audience Participant 2)*

Similarly, a few participants highlighted that the nurses’ experiences depicted through the video resonated deeply with their personal experience of post-traumatic stress incited by the fallout from the pandemic.

*Stressful*,* it’s a flashback. I feel connected somehow as I have a similar experience. (Chat participant 7)*

Feelings of betrayal by those in positions of power and empathetic disappointment was evident among participants post watching the video. Those with a nursing background identified with their colleagues in the monologues as they were trying to manage the unmitigated circumstances to the best of their ability for the benefit of their patients. The overarching agreement from participants was that the video helped their own self-understanding in that the lack of recognition was definitively linked to the lack of support post-COVID which exacerbated the emotional strain they already experienced during the pandemic.

*I think I was sad to hear all that*,* but I’m also very insulted. As a nurse*,* very hurt*,* I think. And that moral injury*,* you know*,* I think for all of us*,* wherever we were*,* we were part of a workforce. We were all trying our best*,* wherever we were*,* to contribute to patient care. (Audience Participant 3)*

The mention of “moral injury” underscored the psychological distress that arose from actions, or the lack thereof, that violated nurses’ moral or ethical code. Most participants indicated that similar to what they watched in the video, nurses felt abandoned and unappreciated, which was influenced by journey of public adoration which moved to a perceived vilification based on the media’s portrayal of the spread of the virus.

“So, what we have seen is from going from the heroes of the hour to the villains of the peace” *(Audience Participant 2)*.

Some participants emphasised that the indignation and anger regarding the lack of support nurses received during COVID-19, has been compounded by the post-Covid pay issues.

…. they clapped Us but don’t want to pay Us! (Survey participant 13)

Post watching the video, participants emphasised the responsibility of managers, they indicated that they resonated with the narrative in the video in that managers’ manner of communication had a major effect on nurses’ sense of work satisfaction and wellbeing. Unhelpful power dynamics and a hierarchy who did not understand what it was like to be directly involved in patient care through the pandemic was a consistent theme stimulated by watching the video.

*Involve managers - they need to see what effect their communications have on morale. We often did the seemingly impossible during the pandemic - still do- to be talked down to and criticised. Like a child coming home with an A and the parent asking’ could you not have done better?’ (Survey participant 17)*.

#### Lessons learned and the need for change

Most participants deemed the video to be an accurate, raw and powerful depiction of the COVID-19 experience for nurses. Post watching the video participants reflected on how nurses were constantly bombarded with rapidly changing information about the virus, its transmission, treatment protocols, and safety guidelines. Many participants who had a nursing background expressed similar feelings of isolation as those presented in the video primarily due to a sense of being misunderstood or underappreciated by the public.

*I think yes very accurate. There was a lot of information that kept changing. Then further into the pandemic public perception changed a lot was out of our control. I felt very isolated (Survey participant 13)*.

The empathetic engagement from participants with a similar professional background demonstrates the effectiveness of the monologues in portraying nurses grappling with the varied challenges of the pandemic without adequate support. In response, participants suggested that dissemination should be wider, most specifically to those in decision making roles and would be useful as part of the NI based COVID-19 inquiry. The most common response indicated that viewers believed narratives like this from the SenseMaker^®^ Report should be televised.

*This video could be part of a TV or radio programme on Covid and how managers failed their staff… the mental and emotional side of what nurses went through*. (*Survey participant* 27)

From most participants, there was a sense of collective responsibility based on the need to turn this research into actionable outputs. Viewers recognised the importance of learning from past experiences and implementing changes to better support nurses and students as a preparatory measure for future crises.

We need to listen, learn and change (Chat participant 9)

While the video captured some aspects of nurses’ experiences during the pandemic, there was a desire among viewers for further research and exploration into the effects of COVID-19 on HCPs in a more varied sectors and a call for a focus on post-pandemic effects. Most strongly, the video inspired an overarching sense of responsibility to use nurses’ stories from the SenseMaker^®^ Report to create positive change for current nurses, future nurses and for the benefit of the wider public accessing the health service.

*They have given us a gift & trusted us with their stories. It is our responsibility to act. We need to change things for our profession and the people we care for* (Survey participant 18).

## Discussion

Overall, the video-based monologues conveyed the emotional effect and lived experiences of nurses during the COVID-19 pandemic, fostering a sense of connection and solidarity among HCPs and the public. The findings evidence interest, involvement and immersion [33] from participants predominantly due to the video’s relevance to the research participants. It is broadly understood that narrative interventions such as video-based monologues are naturally more relevant to human beings than other message formats, for example fact-based messages [34, 35]. In Shaffer’s [33] theoretical model, involvement requires more active participation by the audience than interest. By being involved with a narrative, the audience is able to actively identify with the character [36] and take their perspective, which allows them to experience empathy [37, 38]. As has been noted through the findings, participants unanimously emphasised with the experiences portrayed and those with a nursing background could emotionally identify. Shaffer’s [33] final stage of narrative processing is immersion, which requires a transportation into the narrative and processing incoming information from the perspective of the character involved [34, 38]. The findings confirm that most participants were immersed in the video-based monologues, given the language used “brought me back”, “a flashback”. Although the participants were predominantly part of the nursing community and identification would be expected, the fact that members of the public also deemed the video captivating and the almost unanimous call for wider dissemination signifies that this has relevance to a wider audience.

The findings of this study positively contribute to research within this area. Verbatim narratives such as those gathered through the SenseMaker^®^ report are known to be effective in conveying information due to their ease of understanding and memorability [39, 40]. Similar to the SenseMaker^®^ report, the Humans Not Heroes project began in 2020 in response to the COVID-19 pandemic [41]. In this project, the authors co-created a series of verbatim audio pieces made with artists and healthcare workers exploring their experiences of the Covid pandemic [41]. Although to the best of our knowledge research data on this work has not been published. As far as we know, there is very little research related to the effect of COVID-19 related video-based monologies on a nursing and public audience. The closest study we found of used research-based theatre on the societal impact of COVID-19 [42] similar to our study audience members somewhat/strongly agreed research-based theatre is an suitable means of understanding health research (93.5%) and offered new perspectives on what people had been experiencing (87.5%). Although comparable research is limited, arts-based approaches using verbatim narratives are known to have a unique power in communicating research findings [43–45]. The verbatim approach used in this study ensured that the nurses’ voices were preserved with accuracy and integrity. This method not only honoured the authenticity of their experiences but also underscored the raw and unfiltered nature of their emotions, struggles, and triumphs during the pandemic. The findings highlight that even when these narratives are performed by untrained actors, the emotional resonance remains profound. Despite their lack of formal training, these actors with a nursing background were able to convey the depth of the nurses’ experiences, demonstrating that the power of storytelling lies in the genuine expression of human emotion and connection [43–45]. This study illustrates that when these real-life stories are delivered with sincerity and empathy, the message transcends the need for professional acting skills, resonating deeply with audiences and fostering a greater understanding and appreciation of the nurses’ contributions and sacrifices during COVID-19. Ultimately, this project highlights the important role of narrative in sharing lived experiences, particularly in times of crisis. It showcases how the raw, unpolished recounting of personal stories can evoke strong emotional responses, promote empathy, and provide valuable insights into the human condition, regardless of the storyteller’s training.

The findings collectively highlight that the video meaningful depicted the complex and multifaceted experiences of nurses during the pandemic, including feelings of solidarity, frustration, and the contradictory experience of isolation. This underlies the importance of recognising the powerful role of emotions within the workplace. They underscore the importance of video in raising relevant issues for nurses, this includes the need for social support, effective leadership, and empathy in navigating the emotional challenges inherent in healthcare work, particularly during times of crisis. Like existing evidence [46], the emotional toll of the pandemic on nurses was deeply felt. For example, in the ICON study nurses reported being deeply affected by what they have experienced, and report being forever altered by COVID-19 [10]. It is widely evidenced that the continuous rush of new information created confusion, uncertainty and added stress to HCPs already demanding roles [47]. There was clearly an emotional identification from participants through the memories they recalled depicting the stress of adhering to constantly changing guidelines and protocols while doing their best to ensure the safety of themselves and their patients. Nurses experienced a profound sense of reflection post-viewing the video, highlighting the power of this medium of communication but also the lasting impression of the pandemic on their values, beliefs, and personal lives. Despite the recognition of sacrifices and dedication demonstrated by HCPs, particularly nurses, there was a familiar sense of moral injury and resentment regarding the lack of support and appreciation received post-COVID-19 [48, 49]. Participants highlighted that the video portrayed how nurses were initially acknowledged for their sacrifice and selflessness during the pandemic. However, in response to the video, participants reflected that as the pandemic progressed, the public’s perception of nurses changed. In this context nurses faced increasing scrutiny, scepticism, and even hostility from certain segments of the population. The labelling “nurse as hero” discourse is widely recognised [50], however the stage where politicians, the mass media and the public doubted the quality of HCPs provision of front-line care to people with COVID-19 is under-recognised and under-researched. Discussion of moral injury in the context of the duty to care in reflection of COVID-19 and similar situations warrants future research [48] and dedicated training in self-care strategies [49]. Overall, post watching the video participants concluded that nurses were not adequately supported, undervalued and unappreciated, which inflicted more damage on their morale and sense of value than the pandemic itself.

Following watching the video, participants almost unanimously agreed that emotional effect experienced by nurses during and after the pandemic underscores the need for policy and practice changes to prioritise the emotional well-being of HCPs. The playing back of narratives such as these should reach policy makers to inform policy on embedding staff support in the workplace on an ongoing basis. This resonates with the findings from other studies [51] which focus on the ramifications of burnout for nurses before and during the pandemic and recommend the long-term clinical and preventive psychological interventions which should not be limited to emergencies but extended to address the ongoing challenges faced by nurses. Furthermore, as Connolly et al. state it is of paramount importance that nurses are not blamed for experiencing workplace stress particularly when expressing what is deemed to be normal and appropriate reactions to the extreme circumstances and context of the COVID-19 pandemic [52]. In reflection of the video-based monologues, participants emphasised the importance of validation, recognition, and support for nurses’ emotions within the nursing profession. Similarly, the need for policy and practice change in this realm is ratified by an analysis of mental health effects among nurses working during the COVID-19 pandemic [53] which indicated a prevalence of moderate-to-severe symptoms for anxiety 29.55%, depression 38.79%, posttraumatic stress disorder 29.8%, and insomnia 40.66%. This is particularly relevant given that current literature suggests that experts in policy-making are increasingly recognising the necessity of incorporating narrative as a vital part of the comprehensive evidence base needed to inform complex policy-making processes [54, 55]. Given that policy decisions are often driven by values and politics [56], concise, engaging, and pertinent narratives such as those used in this co-created video would be particularly relevant to current health policy debates [55, 57, 58]. The sensitisation of policy makers to the lived experience of nurses is important in designing sustainable organisational interventions into the future. As most participants emphasised the need for wider broadcasting of the video, particularly to decision-makers in nursing management and policy, more research and targeted dissemination strategies are warranted. Overall, raising awareness and driving positive change through these video-based monologues is validated to preserve and honour the sacrifices made during the pandemic and ensure that policy and practice changes are implemented to better support HCPs and students in future crises.

### Limitations

This study has several limitations. We did not use thematic, content or descriptive analysis of the SenseMaker^®^ report when generating themes or selecting quotes for the video content. However, the process followed reputable co-design principals and methodology [19, 20]. Furthermore, second checking (to reduce the potential for selection bias) was adhered to using two members of the team who were both nurses and very familiar with the report to ensure the quotes were authentically representative. Despite efforts to circulate widely in all possible social media platforms, wider participation was expected. Missing data from demographic section of the survey was also a limitation. The primary limitation concerns the representativeness and generalisability of the sample. Most of the sample were from Northern Ireland which limits application globally and were registered nurses which limits generalisation to the public. The anonymous quotes from audience participation included in the results (from the chat function of the live event and audio recordings) could not be delignated per grouping (nurse / member of the public). The survey was limited to those who had access to a smart phone device, laptop or personal computer and required Wi-Fi signal. This potentially limited access by socioeconomic class able to access the survey. Those with lower socioeconomic class may have bene disproportionately affected and their voices should be included in future research in this area. Despite these limitations, we believe the methodology was the best option available to gauge the effect of the video via the various mediums previously stated.

## Conclusion

The stories generated by nurses in this video provide a record of the SenseMaker^®^ report’s portrayal of how nurses rose to the challenge amidst the chaos, terror and uncertainty of the pandemic. They also help to tell the tale of how nurses shaped the science and trajectory of the pandemic, in many cases innovating in their practice at times when the central therapeutic intervention was nursing care. This work suggests that incorporating narratives in a co-designed nursing video about the COVID-19 experience resulted in successive layers of narrative engagement (e.g. interest, identification, and immersion) making the content relatable across different literacy levels, expertise, and cultures. Further research is needed to assess the broader effect of such innovative methodologies in healthcare-related research. Additionally, further co-created video monologues that utilise more data from the SenseMaker^®^ report would expand upon nurses’ experiences across various sectors during the pandemic. These narratives should be disseminated to a wider audience, amplifying the voices of nurses and highlighting their invaluable contributions during these challenging times. Further screenings and work will provide further opportunities to co-design next steps to influence the public perception and self-identity of nurses not only during the pandemic but have an enduring legacy of empowerment into the future.

## Electronic supplementary material

Below is the link to the electronic supplementary material.

Supplementary Material 1

## Data Availability

The datasets generated and analysed during the current study are not publicly available due to ethical requirements but queries regarding the dataset or analysis can be directed to the corresponding author on reasonable request.

## Abbreviations

COVID-19: Coronavirus disease 2019
HCPs: Health Care Professionals
ICON: Impact of COVID-19 on nurses
MHLS: Faculty of Medicine, Health and Life Sciences
NI: Northern Ireland
QUB: Queens University Belfast
RCN: Royal College of Nursing
STC: Systematic Text Condensation
UK: United Kingdom
W1: Workstream 1
W2: Workstream 2
