# Supplementary material for: Visualising the voices of nursing: a co-designed video capturing the lived experiences of nurses in Northern Ireland during the COVID-19 pandemic
Source: BMC Nurs. 2025 Mar 3;24:237. doi: 10.1186/s12912-025-02881-9 (PMC11877737; doi:10.1186/s12912-025-02881-9)
Supplement: Supplementary file 1 — Supplementary Material 1 [file 12912_2025_2881_MOESM1_ESM.docx]

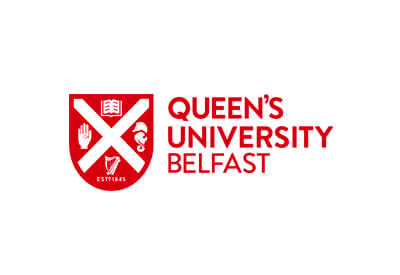


**Title:** Exploring the impact of a co-designed video based on the findings of SenseMaker: the lived experience of nursing in Northern Ireland during a pandemic 2020/2021.

Thank you for reading the information sheet, as noted, submission of a completed online survey will be taken as consent. Thank you for taking time to participate in this online survey.

**Part 1: Demographic information about you, your work setting and role.**

1. Age (Please tick)
   - 18 - 30
   - 31 - 43
   - 44 – 56
   - 57 +
2. Gender (Please tick)
   - Male
   - Female
   - Non-binary
   - Other
3. What is your country of origin?

_______________________

1. Where do you currently work?

- I am a member of the public, not a healthcare professional (please go to question 9)
- Hospital e.g., in-patient, outpatient
- Community e.g., GP practice
- Care home
- Other (please specify) _____________

1. Please specify your background, as a healthcare professional are you a:
   - Nurse
   - Doctor
   - Physiotherapist
   - Dietitian
   - Occupational therapist
   - Speech & language therapist
   - Pharmacist
   - Care worker
   - Other (please specify) ____________
2. How many years have you been qualified in your profession? _______ year (s)
3. Did you work through the pandemic?

- Yes
- No (please go to question 10)

1. Were you redeployed?

- Yes (please go to question 10)
- No (please go to question 10)

1. As a member of the public, please specify your background:
   - I have a family member/friend who is a nurse
   - I was a nurse, I’m now retired
   - I am a patient and I am aware of the pressure nurses are under
   - I had COVID- 19 through the pandemic and was aware of the impact on nurses
   - Other, please specify: _______________

**Part 2: Your experience of the online video**

1. For you, what were the most important benefits of watching the video? Please explain.

__________________________________________________________________________

1. Has your understanding of the emotional impact of the COVID-19 pandemic for nurses changed as a result of watching the video? Please explain.

___________________________________________________________________________

1. In your opinion did the video capture the lived experience of nurses during the COVID-19 pandemic? Please explain.

____________________________________________________

1. Is there anything you expected to see relating to the lived experience of nurses during the COVID-19 pandemic which was absent from the video? Please explain.

__________________________________________________________________________

1. What do you think the next practical steps should be in response to hearing the lived experience of nurses through this video? Please explain.

________________________________________________________________________

1. Who do you think is an appropriate audience for this video? Please explain.

_____________________________________________________________________

1. What do you think should be the next practical steps in regard to disseminating the video to a wider audience? Please explain.

______________________________________________________________________

18. Is there anything else you would like to share?

_________________________________________________________________________

**End of Survey – Thank you for taking the time to complete**
